# Single-atom catalysts for CO_2_ electroreduction with significant activity and selectivity improvements†
†Electronic supplementary information (ESI) available. See DOI: 10.1039/c6sc03911a



**DOI:** 10.1039/c6sc03911a

**Published:** 2016-09-19

**Authors:** Seoin Back, Juhyung Lim, Na-Young Kim, Yong-Hyun Kim, Yousung Jung

**Affiliations:** a Graduate School of EEWS , Korea Advanced Institute of Science and Technology (KAIST) , 291 Daehakro , Daejeon 34141 , Korea . Email: ysjn@kaist.ac.kr; b Graduate School of Nanoscience and Technology , Korea Advanced Institute of Science and Technology (KAIST) , 291 Daehakro , Daejeon 34141 , Korea

## Abstract

A single-atom catalyst (SAC) has an electronic structure that is very different from its bulk counterparts, and has shown an unexpectedly high specific activity with a significant reduction in noble metal usage for CO oxidation, fuel cell and hydrogen evolution applications, although physical origins of such performance enhancements are still poorly understood. Herein, by means of density functional theory (DFT) calculations, we for the first time investigate the great potential of single atom catalysts for CO_2_ electroreduction applications. In particular, we study a single transition metal atom anchored on defective graphene with single or double vacancies, denoted M@sv-Gr or M@dv-Gr, where M = Ag, Au, Co, Cu, Fe, Ir, Ni, Os, Pd, Pt, Rh or Ru, as a CO_2_ reduction catalyst. Many SACs are indeed shown to be highly selective for the CO_2_ reduction reaction over a competitive H_2_ evolution reaction due to favorable adsorption of carboxyl (*COOH) or formate (*OCHO) over hydrogen (*H) on the catalysts. On the basis of free energy profiles, we identified several promising candidate materials for different products; Ni@dv-Gr (limiting potential *U*_L_ = –0.41 V) and Pt@dv-Gr (–0.27 V) for CH_3_OH production, and Os@dv-Gr (–0.52 V) and Ru@dv-Gr (–0.52 V) for CH_4_ production. In particular, the Pt@dv-Gr catalyst shows remarkable reduction in the limiting potential for CH_3_OH production compared to any existing catalysts, synthesized or predicted. To understand the origin of the activity enhancement of SACs, we find that the lack of an atomic ensemble for adsorbate binding and the unique electronic structure of the single atom catalysts as well as orbital interaction play an important role, contributing to binding energies of SACs that deviate considerably from the conventional scaling relation of bulk transition metals.

## Abstract 2

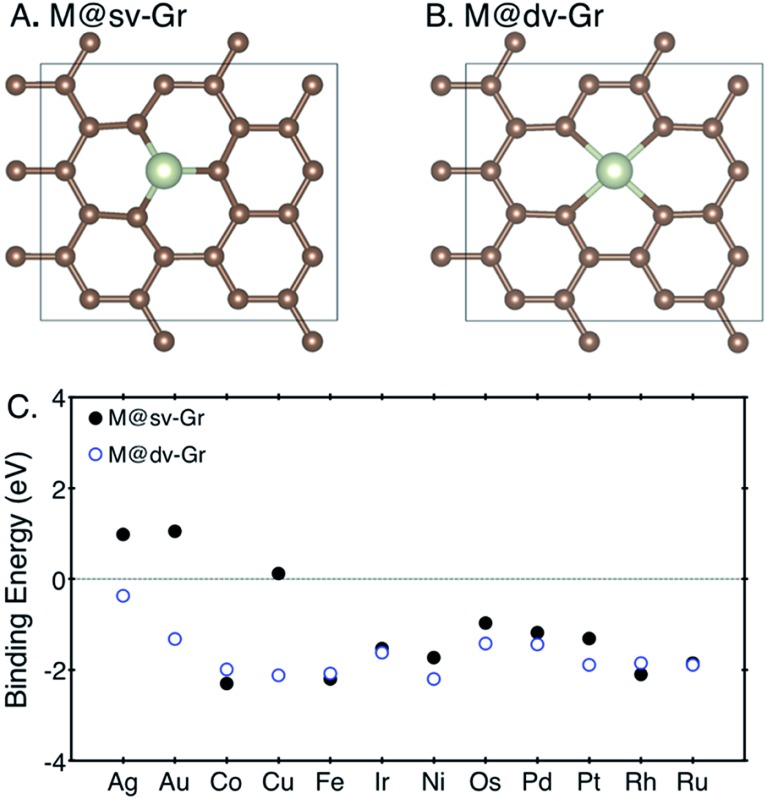
We propose the great potential of single atom catalysts (SACs) for CO_2_ electroreduction with high activity and selectivity predictions over a competitive H_2_ evolution reaction. We find the lack of an atomic ensemble for adsorbate binding and unique electronic structure of the single atom catalysts play an important role.

## 1. Introduction

Due to a limited reservoir of fossil fuels and an increase in atmospheric CO_2_ concentration, there is an urgent need to develop a renewable solution to convert waste CO_2_ into valuable chemicals and fuels. Considerable efforts have been devoted to electrochemical reduction of CO_2_ since this method operates at ambient and mild conditions and can potentially produce various useful hydrocarbons.^1,2^ Transition metal catalysts have been extensively investigated both theoretically and experimentally,^3–9^ and general understanding at present is that a strong correlation between binding energies of various reaction intermediates on transition metals and a lack of ability to independently control them poses a significant intrinsic limitation in developing suitable catalysts for large-scale commercialization of the CO_2_ reduction reaction. For example, for efficient production of CO, the binding energy of *COOH (* meaning adsorbed species on the catalysts) should be strong for facile activation of CO_2_, whereas the binding of *CO and *H should be weak for easy desorption of products and to suppress the unwanted hydrogen evolution reaction (HER), respectively. However, due to a well-known scaling relation, the binding behaviors of *H, *COOH, and *CO all have the same tendency for transition metals in general.^3,7^ Therefore, there is a great interest in developing strategies for deviating from the scaling relation to achieve high selectivity and activity for the CO_2_ reduction reaction (CRR).

Recently, single-atom catalysts (SACs) have been investigated as a promising type of catalyst for various reactions as they surpass conventional catalysts in terms of having a high specific activity with a significantly reduced amount of noble metals used.^10–16^ Pt_1_/FeO_*x*_ was firstly synthesized and utilized as a single atom catalyst for CO oxidation with extremely high activity and stability.^17^ Recently, a single Ni atom was successfully doped at lattice defects of graphene and showed exceptional activity for electrochemical hydrogen production^12^ due to electronic interactions^18^ between the metal and graphene. SACs also offer an intriguing opportunity to alter product selectivity. Single-atom Pt deposited on TiN produced H_2_O_2_ (2 e^–^ pathway) as a major product over H_2_O (4 e^–^ pathway) during the electrochemical O_2_ reduction reaction.^13^

In this work, we investigate a series of single transition metal atoms anchored on defective-graphene as CO_2_ electroreduction catalysts, and report a few catalysts that exhibit a remarkable reduction in overpotentials for CH_3_OH and CH_4_ production. In particular, Pt@dv-Gr is identified as a promising candidate for CH_3_OH production with a substantially reduced limiting potential. The origin of the unusually low overpotentials for SACs is understood using the lack of an atomic ensemble for adsorbate binding and specific metal–support interactions that break the scaling relation for SACs.

## 2. Computational details

Structure relaxation and density of states (DOS) calculations were performed using spin-polarized density-functional theory (DFT) calculations implemented in the Vienna Ab initio Simulation Package (VASP)^19,20^ with projector-augmented wave (PAW) pseudopotential.^21^ Calculated spin moments are summarized in ESI note 1.† We used the RPBE exchange functional,^22,23^ and the van der Waals (vdW) correction^24^ which were previously shown to yield a good agreement with experiments in terms of energetics and electronic structure for similar gas adsorption on an organometallic system.^25^ A cut-off energy for the plane wave basis set was set to 500 eV and *k*-points were sampled using a 4 × 4 × 1 Monkhorst–Pack mesh.^26^

**Fig. 1:**
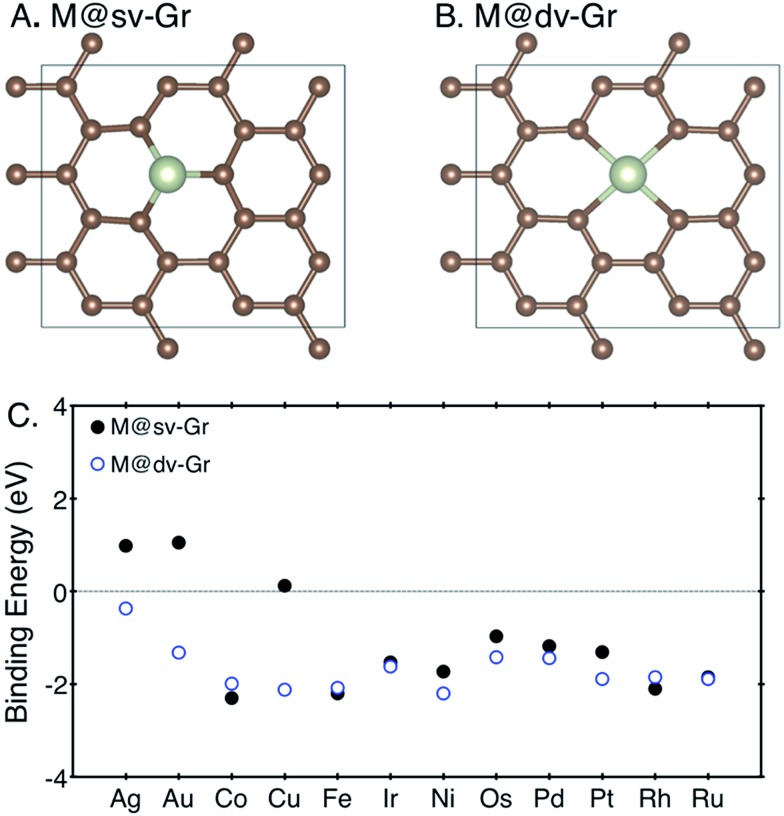
The top view of (A) M@sv-Gr and (B) M@dv-Gr, and (C) binding energies of various transition metal atoms with the sv-Gr (filled black circles) and dv-Gr (open blue circles) defective sites.

To determine the most stable configuration of a metal atom–graphene complex, we compared binding energies of a metal atom on graphene with single or double vacancies. Binding energies of each metal atom at defective graphenes are calculated following the expression *E*_B_[M] = *E*_M/Gr_ – *E*_Gr_ – *E*_M_, where *E*_M/Gr_, *E*_Gr_ and *E*_M_ denote the calculated electronic energies of the metal–graphene complex, defective graphene and metal atom referenced to their metallic states (fcc, bcc, and hcp), respectively. To model the defective graphenes, we firstly used a periodic supercell containing 24 carbon atoms with a vacuum set to 15 Å in a *z*-direction, and then one or two carbon atoms were removed to create the single (sv) or double vacancies (dv) and to provide a site for metal adsorption, referred to as “M@sv-Gr” or “M@dv-Gr”, respectively (Fig. 1A and B). Various transition metal atoms (M = Ag, Au, Co, Cu, Fe, Ir, Ni, Os, Pd, Pt, Rh and Ru) were anchored at the vacant sites of the graphene. We note that our computational unit cell enables us to efficiently calculate various reaction intermediates and it represents high coverage of a single metal atom.

A computational hydrogen electrode (CHE) was used to establish a free energy profile for electrochemical reduction reactions, as pioneered by Nørskov and co-workers.^27^ The limiting potential (*U*_L_) of the reaction is obtained from the free energy change (Δ*G*_MAX_) by using the relation *U*_L_ = –Δ*G*_MAX_/e. For additional calculation details, we referred to our previous publications.^3,4^ Briefly, to convert electronic energies to free energies, zero-point energy, enthalpy and entropy corrections of adsorbates were calculated using a harmonic oscillator approximation at 298.15 K. For molecules, free energy corrections are taken from ref. 6. We also employed an approximate solvation correction to account for the effect of water, where *COOH, *CO and *OH are stabilized by 0.25, 0.1 and 0.5 eV, respectively.^6^ All correction values can be found in the ESI (Table S2).†

## 3. Results

### 3.1. Adsorption of metal atoms at the vacancy site of graphene

For SACs to maintain their catalytic activity for long-term uses, strong binding of a metal atom with a support is a prerequisite to prevent aggregation of metal atoms. Weak binding energies imply that the metal atoms are prone to diffusion with low diffusion barriers, resulting in aggregation to form metal nanoclusters, which usually happens when the metal atom is adsorbed on defect-free graphene.^28,29^ We thus first calculated the binding energies of the metal atoms at the sv-Gr or dv-Gr sites, and the results are summarized in Fig. 1C. Most of the metals have favorable and sufficiently strong binding with the aforementioned defect sites of graphene. In detail, for Co, Fe and Rh, the sv-Gr binding is more favorable than the dv-Gr binding, but for all other cases, the dv-Gr binds the metals more strongly. Therefore, for the rest of this paper, we will consider CO_2_ electrochemical reduction reactions for M = Co, Fe and Rh using the M@sv-Gr model, and M = Ag, Au, Cu, Ir, Ni, Os, Pd, Pt and Ru using the M@dv-Gr model. In conjunction with the strong binding energies of the metal atoms, Bader charge analysis^30,31^ indicates that, as expected, significant amounts of electrons in the metal atoms are transferred to the graphene in all cases, confirming the strong covalent interactions between the partially positively charged metal atom and the graphene (Table S1†).^29,32^

### 3.2. CO_2_ electroreduction reaction on the SACs

#### 3.2.1. Initial protonation steps: selectivity for CRR *vs.* HER

**Fig. 2:**
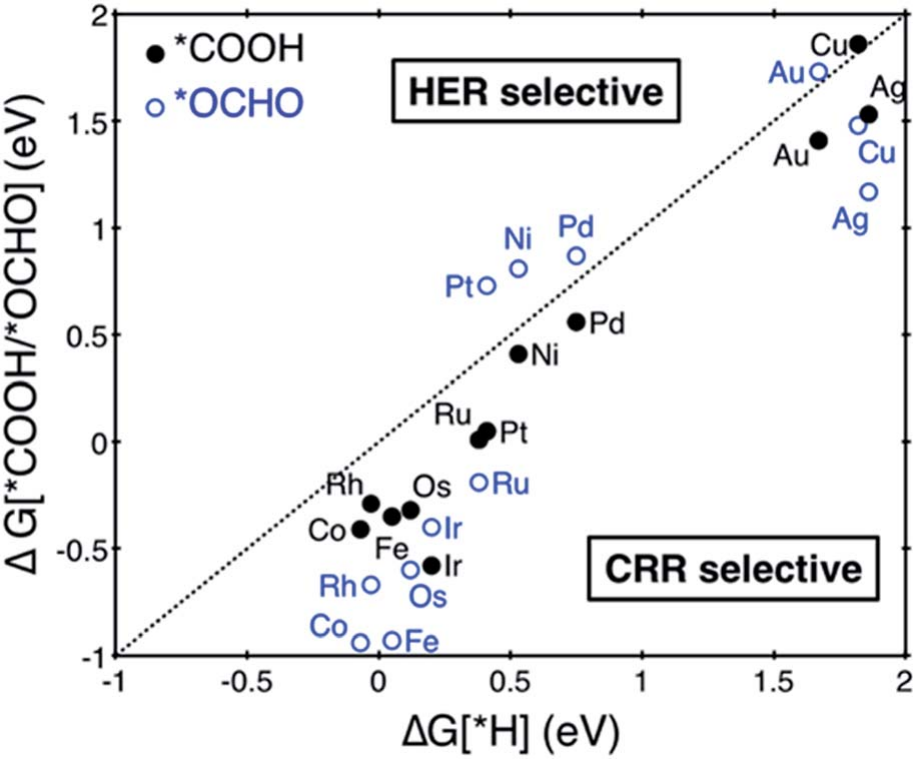
Free energy change of the first protonation step in the CO_2_ reduction reaction (CRR) and H_2_ evolution reaction (HER) on the various SACs. Catalysts below the dotted parity line are CRR selective.

**Fig. 3:**
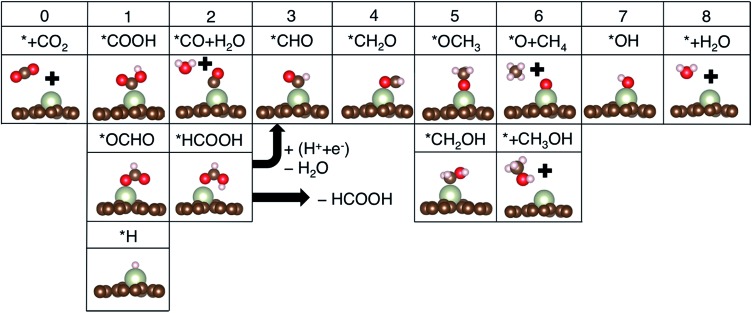
Key reaction intermediate species for the CO_2_ reduction reaction. The numbers on top of each column are the numbers of proton–electron pairs (H^+^ + e^–^) transferred to CO_2_.

The CO_2_ electroreduction reaction (CRR) begins by protonation of CO_2_ to form either adsorbed carboxyl (*COOH) or formate (*OCHO) on the catalysts (see Fig. 3 to compare the two binding configurations *COOH *vs.* *OCHO). Under the same reaction conditions, *H may also adsorb on the catalysts by consuming the same proton–electron pair (H^+^ + e^–^) and undergo a potentially unwanted HER side reaction. We thus first compared free energy changes (Δ*G*) of three initial protonation steps, formation of *COOH, *OCHO and *H. Based on the Brønsted–Evans–Polanyi relation, which correlates reaction barriers with reaction energies,^33,34^ we assumed that reactions with lower free energies are more selective. As seen from the results summarized in Fig. 2, most SACs are below the parity line that determines the selectivity for CRR *vs.* HER, meaning that it is more favorable to form either *COOH or *OCHO than *H, and single metal sites can be effectively utilized for CRR rather than HER. Possible reaction pathways for producing HCOOH, CH_3_OH and CH_4_ are shown in Fig. 3. In this work, only mono-carbon products are considered to form on the SACs since the lack of a metal ensemble in single metal catalysts is expected to prevent C–C coupling reactions between reaction intermediates to form multi-carbon adducts.

#### 3.2.2. Production of HCOOH, CH_3_OH and CH_4_

We next considered protonation of *COOH or *OCHO by evaluating free energies of further reduction intermediates. For SACs following *COOH pathways, the protonation of *COOH produces *CO. This *CO could then either desorb off from the catalyst surface or be further protonated to *CHO depending on the relative free energy changes of desorption (*CO → * + CO) *vs.* protonation (*CO + H^+^ + e^–^ → *CHO). Similarly, for SACs following the *OCHO pathways, once *OCHO is protonated in the form of *HCOOH, it could either desorb off from the catalyst surface or be further protonated to *CHO depending on the relative free energy changes of desorption (*HCOOH → * + HCOOH) *vs.* protonation (*HCOOH + H^+^ + e^–^ → *CHO + H_2_O). We found that the formation of *COH or *OCH_2_OH in the third protonation step is energetically less favorable than the formation of *CHO in all cases.

Additional protonation of *CHO can form either *OCH_3_ (known to determine the selectivity between CH_4_ and CH_3_OH)^4^ or *CH_2_OH (which only yields CH_3_OH upon further protonation since that is more favorable than the formation of *CH_2_ + H_2_O in all cases). Free energy profiles for a few promising candidate materials are summarized in Fig. S1,† and the potential determining step (PDS) and limiting potential (*U*_L_) toward the most favorable products are summarized in Table 1.

**Table 1:** The calculated potential determining steps (PDS) and limiting potentials (*U*_L_, V) for the production of HCOOH, CH_4_ and CH_3_OH

Metal	PDS	*U*_L_
Ag^*a*^	CO_2_ → *OCHO	–1.17
Au^*a*^	CO_2_ → *COOH	–1.41
Co^*b*^	*OCHO → *HCOOH	–0.56
Cu^*c*^	CO_2_ → *OCHO	–1.48
Fe^*b*^	*HCOOH → *CHO	–0.73
Ir^*c*^	*CH_2_OH → CH_3_OH	–0.57
Ni^*c*^	CO_2_ → *COOH	–0.41
Os^*b*^	*OH → *+H_2_O	–0.52
Pd^*c*^	*CO → *CHO	–0.62
Pt^*c*^	*CO → *CHO	–0.27
Rh^*c*^	*OCH_3_ → CH_3_OH	–0.57
Ru^*b*^	*HCOOH → *CHO	–0.52

^*a*^HCOOH production.

^*b*^CH_4_ production.

^*c*^CH_3_OH production.

**Fig. 4:**
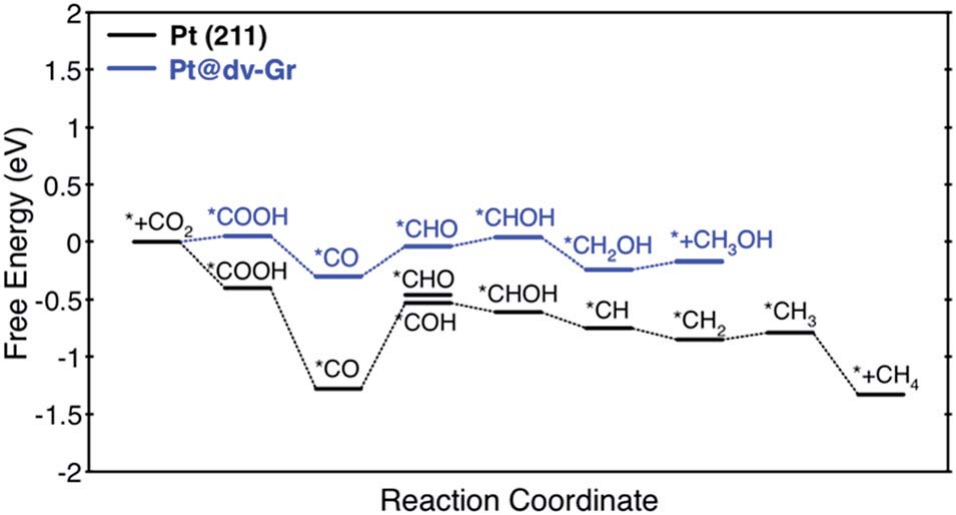
Free energy profiles for the CO_2_ reduction reaction to CH_4_ and CH_3_OH on Pt (211) and Pt@dv-Gr, respectively, at zero applied voltage (*vs.* RHE). Free energy changes of the PDS for Pt (211) and Pt@dv-Gr are 0.75 eV (*CO → *COH) eV and 0.27 eV (*CO → *CHO), respectively.

Among the considered SACs, Ni@dv-Gr and Pt@dv-Gr showed a *U*_L_ of –0.41 and –0.27 V, respectively, for CH_3_OH production, while Os@dv-Gr and Ru@dv-Gr both showed a *U*_L_ of –0.52 V for CH_4_ production. To put these theoretical predictions into perspective, it is useful to make links to previous theoretical and experimental results. For example, the theoretical limiting potentials for CH_4_ production on Ni (211), Pt (211) and Cu (211) surfaces are –0.7 to –0.8 V *vs.* RHE.^4^ However, in an experiment for the CO_2_ reduction reaction, polycrystalline Ni and Pt produced H_2_ gas as a major product almost exclusively and only a trace of CH_4_ with a total current density of 5 mA cm^–2^ at –1.08 V and –0.67 V *vs.* RHE, respectively, while Cu produced CH_4_, C_2_H_4_, and H_2_ in similar amounts with a total current density of 5 mA cm^–2^ at –1.05 V *vs.* RHE.^35^ It is thus noteworthy that the experimental product selectivity is significantly different for Ni, Pt and Cu, even though their theoretical limiting potentials for the CO_2_ reduction reaction are similar. The origin of the dominant H_2_ production during the CO_2_ reduction reaction has been suggested to be that for strong *CO binding catalysts such as Ni and Pt, the *H binding energies shift toward being weaker as the coverage of *CO increases due to a repulsive interaction between *CO and *H. This then makes Ni and Pt catalysts more active for the HER (shifting from the left leg to the top of a HER volcano, therefore reducing the limiting potentials for the HER).^8,36^ On the other hand, the increasing *CO coverage negatively affects the HER on moderate or weak *CO binding catalysts such as Cu (shifting more to the right leg of the HER volcano, therefore increasing the limiting potential for the HER). Therefore, to achieve high activity and selectivity for the CO_2_ reduction reaction, the catalysts should have less negative limiting potentials and bind *CO moderately at the same time if the coverage effects are expected to be considerable. However, since all the active sites of the SACs are isolated, we suppose that there are no such coverage effects as in conventional metal catalysts, which leads to the conclusion that the aforementioned SACs can produce the desired reduction products over H_2_ with considerably reduced limiting potentials. In particular, as in the free energy diagram shown in Fig. 4 in detail, the predicted theoretical limiting potential (*U*_L_) for CH_3_OH production using Pt@dv-Gr is significantly less negative than any catalysts that produce CH_4_ or CH_3_OH in the literature, synthesized (–0.5 to –1.0 V)^35,37,38^ or predicted (–0.3 V to –1.0 V).^4,6,39,40^

### 3.3. Origin of large activity improvement on the SACs

Here, we focus on Pt@dv-Gr to investigate the origin of the activity improvement on SACs compared to their transition metal counterparts. Notably, as shown in Fig. 4, the PDS of CO_2_ reduction on Pt@dv-Gr *vs.* Pt (211) is the protonation of *CO to form *CHO (Δ*G* = 0.27 eV) *vs.* *COH (Δ*G* = 0.75 eV). In Fig. 4, it is visually clear that, with the Pt@dv-Gr catalyst, all reaction intermediates are destabilized compared to those on Pt (211), but most importantly, the destabilization of *CO (0.98 eV) is much more noticeable than that of *CHO (0.42 eV), leading to a 0.49 V reduction in the limiting potential. Therefore, understanding the origin of the different stabilities of *CO and *CHO (or *COH) on Pt@dv-Gr *vs.* Pt (211) surfaces is key to explaining the activity improvements of the Pt-based SAC. As the free energy of *CHO and *COH on the Pt (211) is similar, we will discuss the relative stabilities of *CO and *CHO in order to directly compare with those on Pt@dv-Gr.

**Fig. 5:**
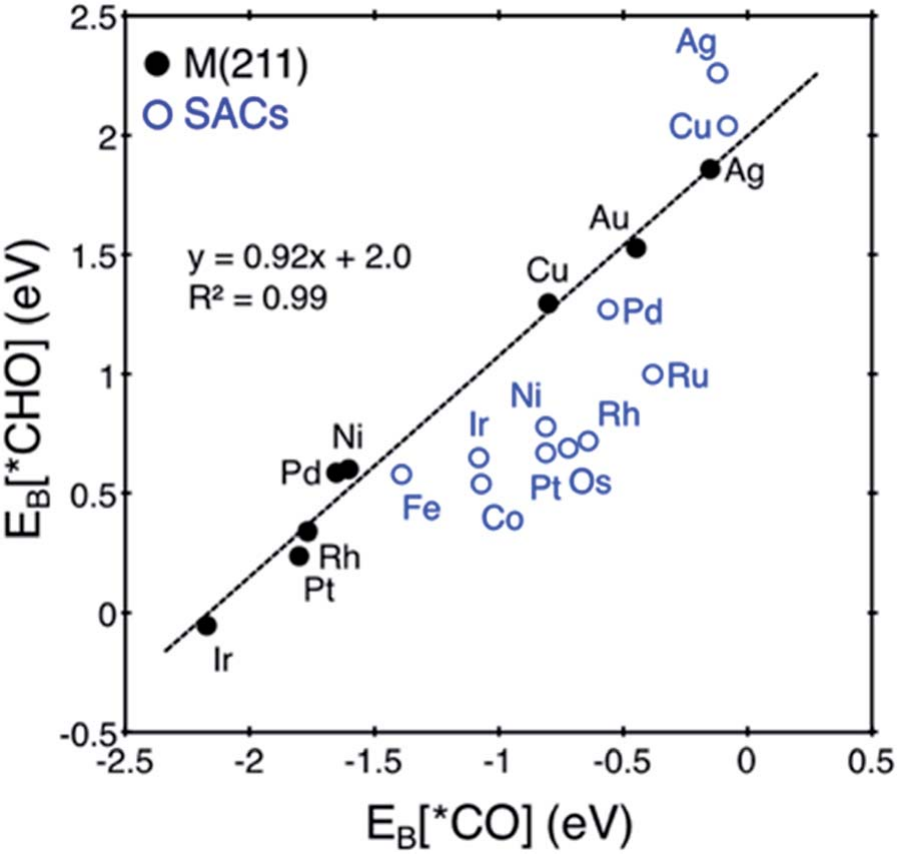
Correlation between *E*_B_[*CO] and *E*_B_[*CHO] for transition metal (211) surfaces (black) *vs.* SACs (blue). The conventional scaling relation for M (211) is broken in the case of SACs. We note that Au@dv-Gr is not shown since it does not bind *CO.

In Fig. 5, we also observe that with SACs, the conventional scaling relation between *CO binding and *CHO binding, that is well established for the bulk transition metal catalysts, significantly deviates from linearity. In the following, we thus discuss features of SACs which contribute to the breakdown of this scaling relation between *CO and *CHO, namely, a lack of atomic ensemble for adsorbate binding and metal–support interactions that lead to electronic structures conducive to catalysis.

#### 3.3.1. Atomic ensemble

**Fig. 6:**
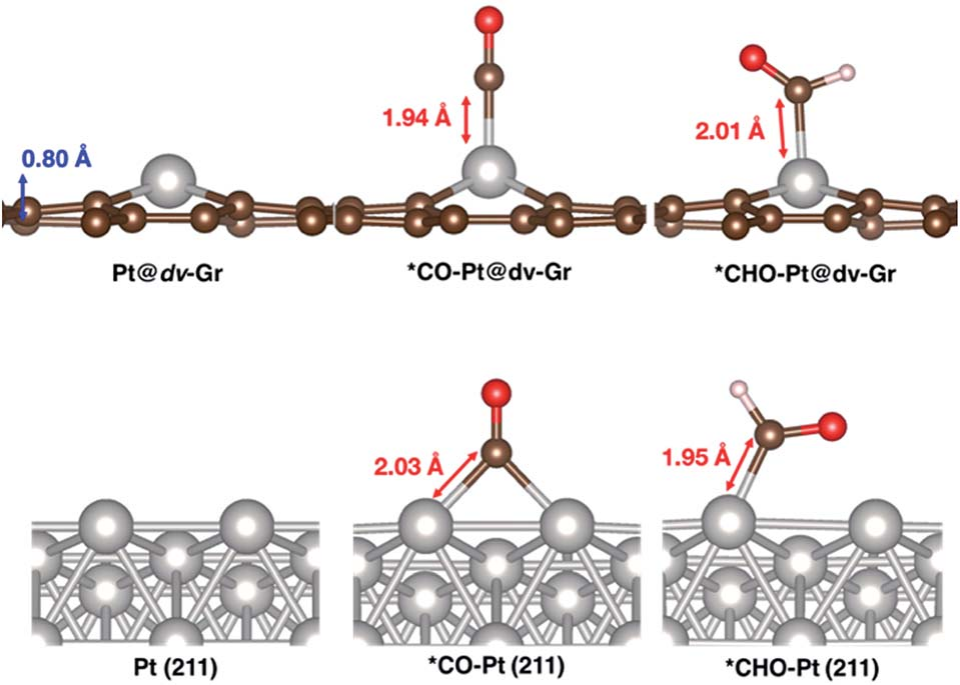
Optimized geometries of Pt@dv-Gr and Pt (211) before and after adsorption of *CO and *CHO.

Optimized geometries of bare catalysts as well as *CO and *CHO adsorbed catalyst surfaces are shown for Pt@dv-Gr and Pt (211) in Fig. 6. One can immediately see that, for Pt (211), two surface Pt atoms are involved in *CO bonding, while only one Pt atom bonds with *CHO upon further protonation, leading to a large destabilization of relative free energies when going from *CO to *CHO. On the other hand, for Pt@dv-Gr, only one Pt atom is utilized by definition for both *CO and *CHO binding, resulting in the much more moderate destabilization of the relative free energies compared to Pt (211). Thus, the lack of a Pt ensemble in Pt@dv-Gr is responsible for significantly weaker binding of *CO on Pt@dv-Gr (*via* one Pt–C bond) compared to the Pt (211) surface (*via* two Pt–C bonds).

#### 3.3.2. Electronic structure of the single atom

A strong metal–support interaction affects the electronic structure of a metal atom in SACs greatly, which then determines catalytic properties of the SACs.^11–13^ As can be seen in Fig. S2A,† the Pt 5d density of states (DOS) in Pt@dv-Gr shows significant orbital overlap with C 2p orbitals of graphene. Electron density isosurfaces (Fig. S2B†) visually illustrate that electron clouds of four carbon atoms surrounding the Pt atom are significantly hybridized with the Pt atom. A differential charge density map (Fig. S2C†) between the defective graphene and Pt@dv-Gr also suggests that the Pt atom is positively charged (oxidized) by electron transfer from the Pt atom to the defective graphene support (0.79 e from Bader population analysis, Table S1†). Below, we show in more detail that this metal–support interaction in the SACs, *i.e.*, mixing of p-orbital contributions from the support material into the d-character of the metal and the resulting charge redistribution of the SAC, is the main origin of the broken scaling relation (Fig. 5) and improved activities (Fig. 4) since the conventional scaling relation originates from the d-band center theory.^41,42^ It was indeed observed that the SACs showed less correlation between the d-band center and *CO binding energies than the metal (211) surfaces (ESI note 2†).

In understanding the poor scaling relation between the *CO and *CHO bindings in SACs (Fig. 5), we focus on Pt and Cu as representative cases since Pt shows a negative deviation (below the usual scaling trend line) and Cu shows a positive deviation (above the trend line).

**Fig. 7:**
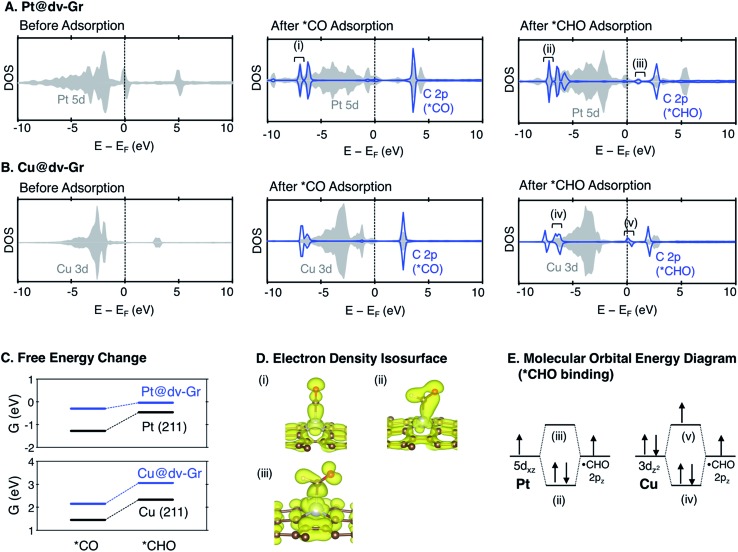
The density of states (DOS) for a metal d orbital and a carbon 2p orbital in adsorbates before adsorption, and after *CO and *CHO adsorption for (A) Pt@dv-Gr and (B) Cu@dv-Gr. (C) Free energy change for the protonation of *CO to *CHO (*CO + H^+^ + e^–^ → *CHO) on SACs and (211) stepped surfaces. (D) The electron density isosurfaces at the energy level as noted with (i), (ii), and (iii) in (A). (E) The schematic molecular orbital energy diagram for *CHO binding on Pt@dv-Gr and Cu@dv-Gr.

As shown in Fig. 7 and S3,† the major bonding interaction between *CO and Pt@dv-Gr is the Pt(d_*z*_^2^)–C(p_*z*_) σ bond along the *z*-direction at around –7 eV (denoted (i) in Fig. 7A). The next sharp peak at around –6 eV corresponds to a covalent C–O bond (denoted (ii) in Fig. S3†) without much mixing with the single metal atom. For *CHO binding, three overlapping peaks are noticeable below the Fermi level. The first sharp peak corresponds to the bonding interaction between C(p_*z*_) and a hybridized orbital of Pt(d_*xz*_) and Pt(d_*z*_^2^) (denoted (ii) in Fig. 7A). The second peak corresponds to the bonding interaction between C(p_*x*_), C(p_*z*_) and Pt(d_*z*_^2^) (denoted (iv) in Fig. S3†). The third peak corresponds to the localized bonding interaction between C and O since the DOS of C does not change upon adsorption (denoted (v) in Fig. S3†).

For Cu@dv-Gr, on the other hand, the orbital contribution for *CO binding is also the σ bonding interactions along the *z*-direction similar to Pt@dv-Gr, yet there is a much weaker overlap than that of Pt@dv-Gr (Fig. S3 and S4†), giving a *CO binding energy on Cu@dv-Gr (–0.08 eV) that is substantially weaker than that on Pt@dv-Gr (–0.81 eV). In addition, a more important feature distinctive of Cu compared to Pt appears in *CHO binding (see Fig. S4†); the orbital (v) in Fig. 7B that is an antibonding counterpart of the σ(Pt(d_*z*_^2^)–C(p_*z*_)) bond ((iv) in Fig. 7B) is partially occupied unlike the similar state that is completely unfilled in Pt ((iii) in Fig. 7A). This partial occupancy of the antibonding orbital in Cu@dv-Gr weakens the metal–*CHO binding strength, and with a lack of such antibonding occupancy for *CO binding, the relative *CO *vs.* *CHO free energy difference that determines the limiting potential significantly increases for Cu@dv-Gr compared to Pt@dv-Gr. This weakening of *CHO binding for Cu@dv-Gr can be schematically understood as in Fig. 7E since the Cu atom in the almost square-planar symmetry has a completely filled d_8_ electron configuration to begin. By contrast, Pt only fills the bonding orbital *via* orbital mixing due to a single occupancy in the d_*xz*_ orbital that can interact with the C(p_*z*_) orbital of *CHO. To further validate this interpretation, we performed the same analysis for Ag@dv-Gr and Au@dv-Gr, and confirmed the same completely filled d_8_-block elements as in Cu. Indeed, we observe that the antibonding states originating from the interaction of metal d_*z*_^2^ and C(p_*z*_) are partially filled (Fig. S5†) in these elements also, leading to the destabilization of *CHO, and eventually the positive deviation from the usual *CO *vs.* *CHO scaling relation shown in Fig. 5. To extend our understanding to other metals, we additionally analyzed the DOS for *CHO adsorption on metals in other SACs (Fig. S7 and ESI note 3†), and it was observed that the antibonding states are partially filled only for metals in group 11 (Ag, Au and Cu).

## 4. Conclusions

In this paper, we investigated single atom catalysts (SACs) as promising CO_2_ electroreduction catalysts using DFT calculations. The main findings of this work are as follows.

(i) By comparing free energies of the initial protonation steps for the CRR and HER, we found that all the candidate SACs are capable of selectively reducing CO_2_ rather than producing hydrogen gas. Among the considered SACs, Ni@dv-Gr and Pt@dv-Gr showed a *U*_L_ of –0.41 and –0.27 V for CH_3_OH production, while Os@dv-Gr and Ru@dv-Gr both showed a *U*_L_ of –0.52 V for CH_4_ production. In particular, the predicted limiting potential for Pt@dv-Gr (–0.27 V) for CH_3_OH production is considerably less negative than for conventional transition metal catalysts (–0.7 to –0.8 V).

(ii) To understand the origin of the activity improvements using SACs, we investigated two aspects (the atomic ensemble and the electronic structure) of Pt@dv-Gr that affect the relative stability of *CO *vs.* *CHO. A one-fold bonding of *CO on Pt@dv-Gr due to a lack of atomic ensemble, as compared to the two-fold *CO bonding on Pt (211), is responsible for the significant weakening of the *CO binding on Pt@dv-Gr.

(iii) We investigated the electronic structure of a Pt atom in the SAC to find the origin of the deviation of SACs from the conventional scaling relation of transition metals, which arises from the d-band center theory. We suggest that the strong electronic interaction between the d-orbital of the metal atom and the p-orbital of graphene is responsible for the different behavior from the transition metal surfaces, as evidenced by the electron transfer and the overlap in the DOS. We particularly noticed a difference in the direction of the latter deviation for Ag and Cu-based SACs *vs.* other SACs. By analyzing the decomposed density of states, we found that the completely filled d_8_ electron configuration leads to the partial occupation of antibonding orbitals during the *CHO binding for the Ag, Au, and Cu based SACs, weakening the *CHO binding and increasing the limiting potential.
