# Supplementary material for: Single-atom catalysts for CO2 electroreduction with significant activity and selectivity improvements
Source: Chem Sci. 2016 Sep 19;8(2):1090–6. doi: 10.1039/c6sc03911a (PMC5369399; doi:10.1039/c6sc03911a)
Supplement: Supplementary file 1 [file SC-008-c6sc03911a-s001.pdf]

Supporting Information for

“Single-Atom Catalysts for CO_2 Electroreduction with Significant Activity and Selectivity Improvements”

Seoin Back[a], Juhyung Lim[a], Na-Young Kim[b], Yong-Hyun Kim[b] and Yousung Jung [a,*]

[a]Graduate School of EEWS and [b]Graduate School of Nanoscience and Technology, Korea Advanced Institute of Science and Technology (KAIST), 291 Daehakro, Daejeon 34141, Korea

Supplementary Note 1: Spin Moment

	Ag (dv)	Au (dv)	Co (sv)	Cu (dv)	Fe (sv)	Ir (dv)	Ni (dv)	Os (dv)	Pd (dv)	Pt (dv)	Rh (sv)	Ru (dv)
Spin Moment (μ_B)	0	0	0.34	0	0	0.46	0	0	0	0.31	0.21	0
$E_{spin} - E_{non\text{-}spin}$			-0.05			-0.04				-0.02	-0.05	

It was reported that the spin moments of metal atoms change significantly depending on the type of vacancy (single or double) and neighboring atoms.[1, 2] For example, the spin moment of Fe atom increases from 0 to 3.16 when the morphology changes from Fe@sv-Gr (in this study) to Fe@sv-N_3-Gr (Fe neighboring three carbon atoms are replaced by nitrogen atoms).[2] In that case, the energy difference between spin-polarized and spin non-polarized solutions was significant (0.5 eV). On the other hand, the spin moments of metal atom adsorbed in the sv-Gr or dv-Gr are very small, and the energy differences between spin-polarized and spin non-polarized solutions for Co@sv-Gr, Ir@dv-Gr, Pt@dv-Gr and Rh@sv-GR were within 0.05 eV, suggesting that the effects of spin are trivial in our case.

Table S1. Bader charge of metal atoms adsorbed on the graphene with single or double vacancies. Positive charges indicate that an electron is transferred from the metal atoms to graphene.

	Bader charge of Metal (e)		Bader charge of Metal (e)
Ag@dv-Gr	0.75	Ni@dv-Gr	0.75
Au@dv-Gr	0.75	Os@dv-Gr	0.30
Co@sv-Gr	0.66	Pd@dv-Gr	0.63
Cu@dv-Gr	0.77	Pt@dv-Gr	0.79
Fe@sv-Gr	0.93	Rh@sv-Gr	0.58
Ir@dv-Gr	0.95	Ru@dv-Gr	1.10

Table S2. The calculated free energy corrections for adsorbates. All values are given in eV.

Adsorbate	Zero Point Energies	$\int C_p\, dT$	-TS
*CH_2O	0.73	0.11	-0.24
*CH_2OH	1.06	0.09	-0.19
*CHO	0.44	0.07	-0.15
*COH	0.39	0.06	-0.13
*COOH	0.59	0.10	-0.22
*CO	0.16	0.07	-0.19
*OCH_3	1.05	0.08	-0.22
*OH	0.32	0.05	-0.10
*O	0.07	0.04	-0.06
*CHOH	0.74	0.09	-0.22
*OCHO	0.58	0.10	-0.22
*OCH_2O	0.78	0.11	-0.26
*HCOOH	0.90	0.07	-0.13
*H	0.16	0.01	-0.02
*CH	0.24	0.05	-0.10
*CH_2	0.56	0.06	-0.11
*CH_3	0.91	0.06	-0.12

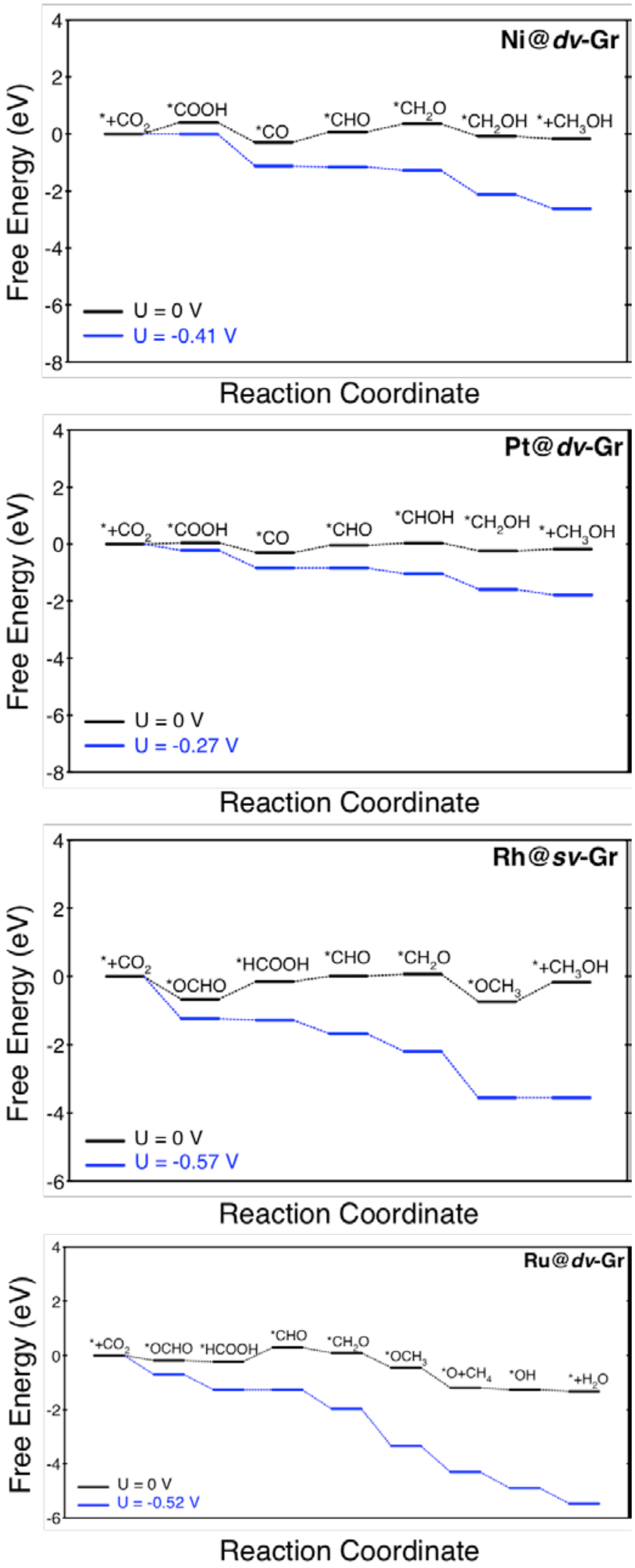

Figure S1. Free energy diagrams for the CO_2 reduction reaction on a few promising candidate materials. Black and blue lines indicate the free energies at 0 V (vs. RHE) and at their limiting potentials, respectively.

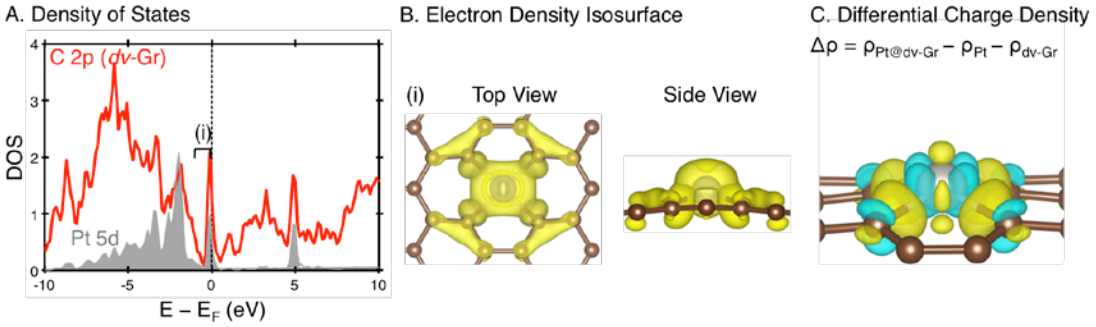

Figure S2. (A) The density of states (DOS) for Pt 5d orbital and C 2p orbital in graphene for the Pt@dv-Gr. (B) Top and side views of the electron density isosurface at the energy level as noted with (i) in A (isosurface level: 0.04 e/Å^3). (C) The differential charge density map upon adsorption of Pt on the dv-Gr. In the differential charge density map, yellow and blue region indicate electron accumulation and depletion, respectively. (isosurface level: 0.04 e/Å^3)

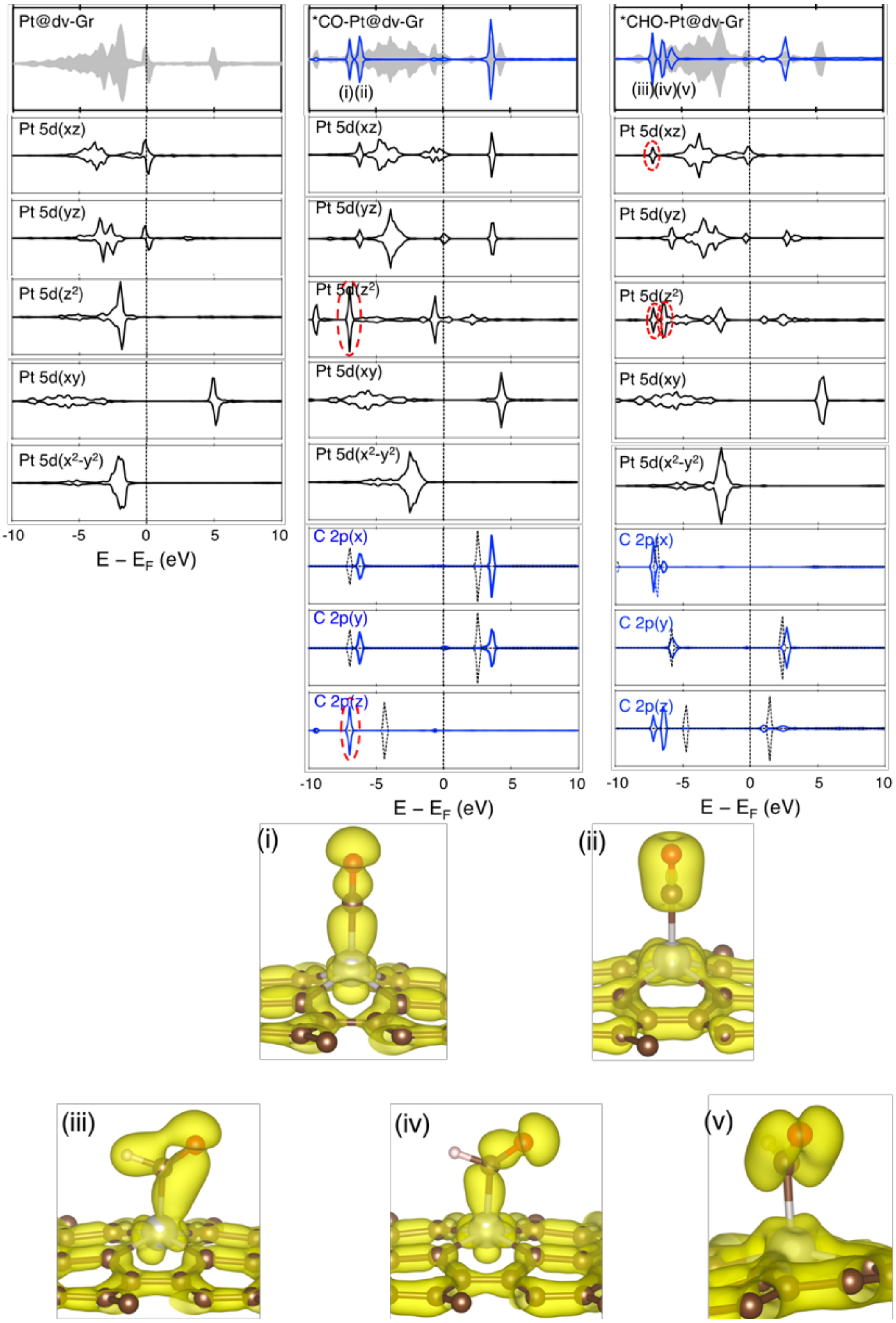

Figure S3. The density of states (DOS) for Pt 5d orbital and C 2p orbital in adsorbates (*CO and *CHO) before and after *CO, *CHO adsorption for the Pt@dv-Gr. The black dashed lines indicate the DOS of desorbed CO and the red dashed circles indicate orbital interactions of interest.

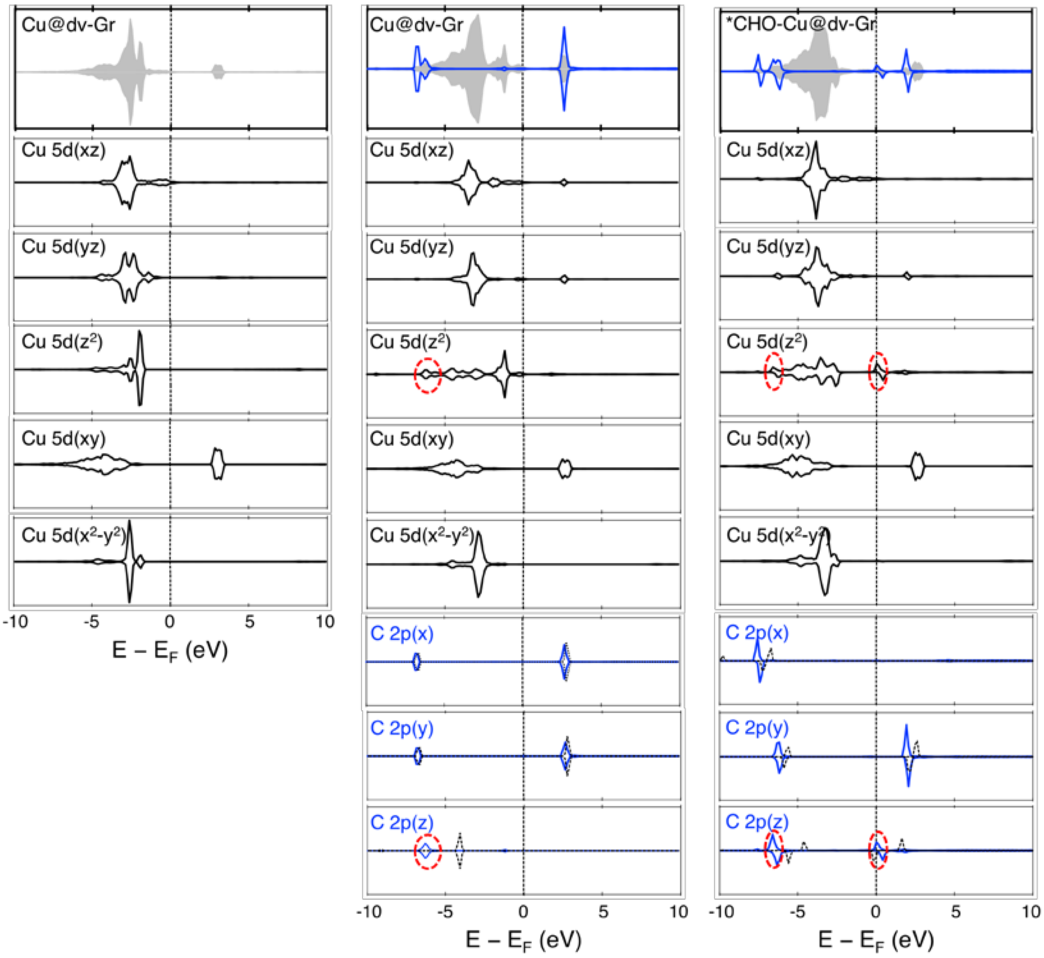

Figure S4. The density of states (DOS) for Cu 3d orbital and C 2p orbital in adsorbates (*CO and *CHO) before and after *CO, *CHO adsorption for the Cu@dv-Gr. The black dashed lines indicate the DOS of desorbed CO and the red dashed circles indicate orbital interactions of interest.

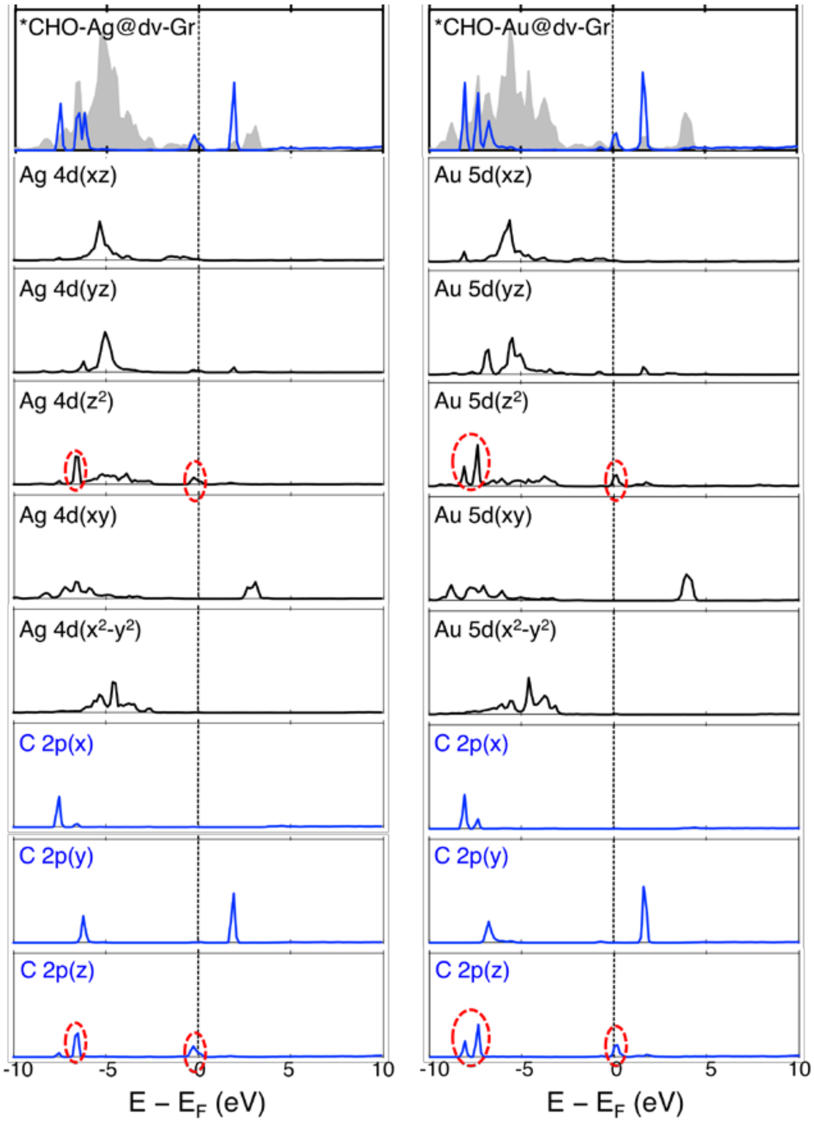

Figure S5. The density of states (DOS) for Ag 4d, Au 5d orbital and C 2p orbital in *CHO for the Ag@dv-Gr and Au@dv-Gr. The red dashed circles indicate filled bonding and corresponding partially filled antibonding states identical to Cu@dv-Gr.

Supplementary Note 2: Comparison between metal (211) surfaces and SACs

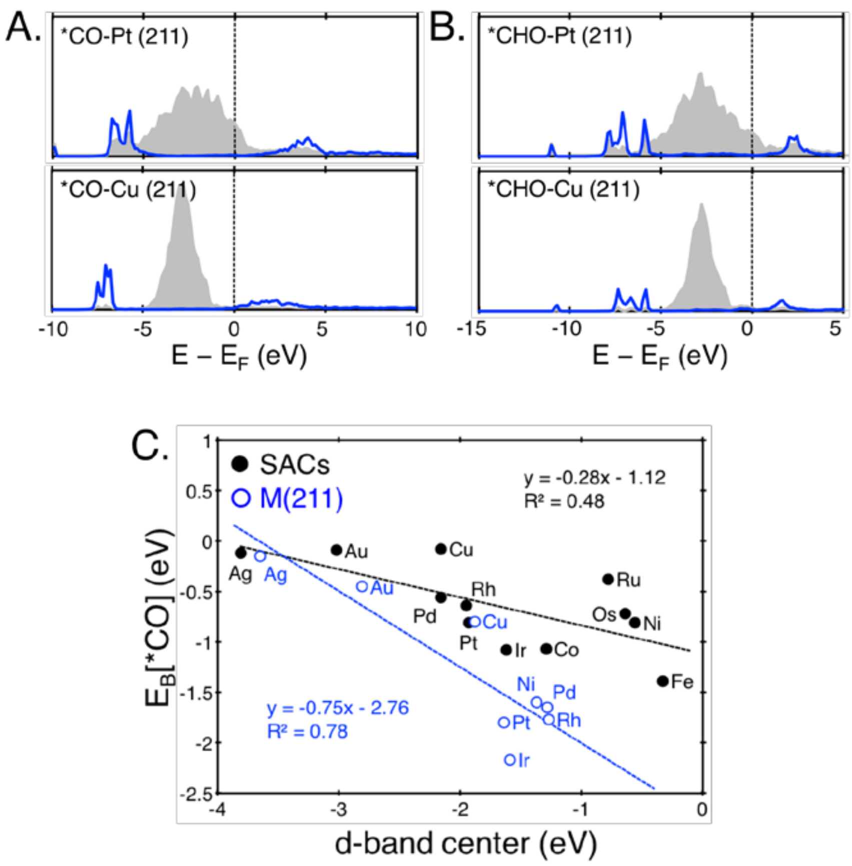

Figure S6. The density of states (DOS) of metal d orbital (grey) and carbon p orbital (blue) for (A) *CO and (B) *CHO adsorption on Cu (211) and Pt (211). (C) Correlation between d-band center and *CO binding energy on metal (211) (open blue circles) and SACs (black circles).

Figure S6 (A) and (B) show DOS for *CO and *CHO adsorption on Cu (211) and Pt (211). It appears that the bonding mechanism for each adsorbate is analogous for different metals. This is, however, not the case for single atom catalysts, where the different bonding mechanism yielded opposite results (below and above the scaling relation for Pt@dv-Gr and Cu@dv-Gr, respectively).

We additionally considered the d-band center, which is usually used as a descriptor for gas adsorption on metal surfaces.[3] We calculated and plotted the d-band center for metal (211) surfaces, which is known to be correlated with the binding affinity. Metal (211) surfaces showed a reasonable correlation between d-band center and *CO

binding energies, while single atom catalysts showed less correlation since, in this case, carbon p orbitals also take part in the binding, breaking the d-band center based scaling relation.

Supplementary Note 3: Discussion on other metal dopants in SACs

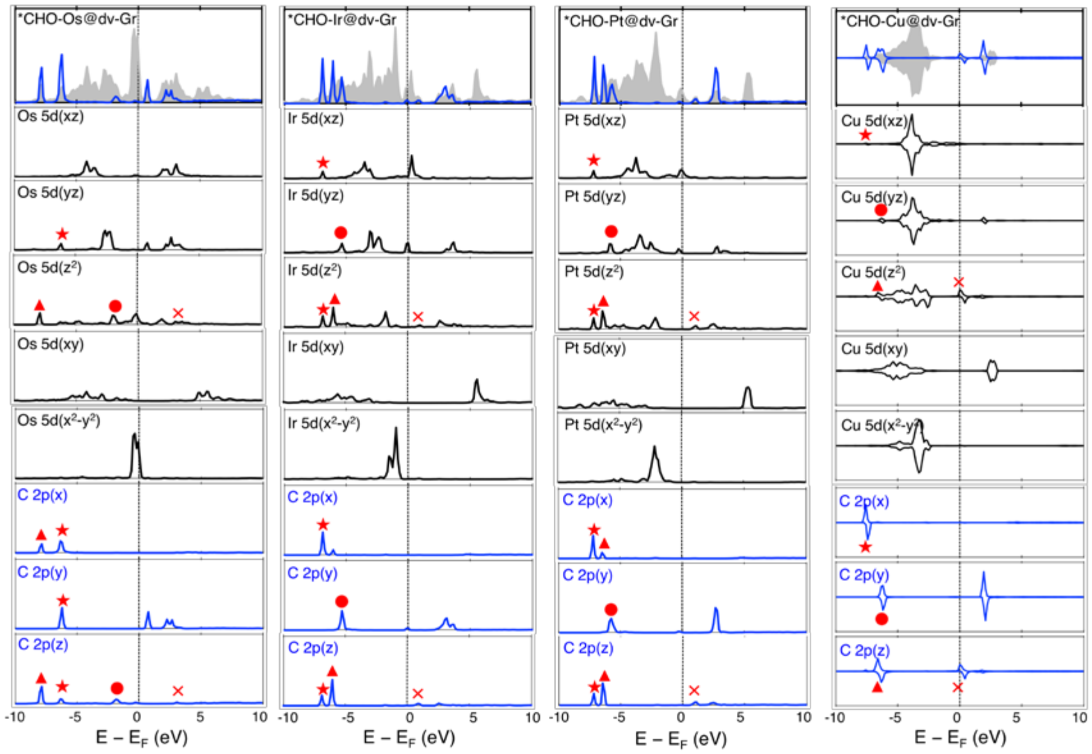

Figure S7. The density of states (DOS) for *CHO adsorption on Os, Ir, Pt and Cu. Triangle, star, and circle marks indicate bonding interactions between metal d orbital and carbon p orbital in *CHO, while cross indicates anti-bonding interaction.

We analyzed the DOS for Os and Ir, and compared the Os, Ir, Pt, and Cu systematically, one metal atom in each group (from group 8 to group 11) as representatives. In Figure S7, one can see that the *CHO binding on Os and Ir also show roughly the similar qualitative behavior as in Pt@dv-Gr that the antibonding orbital, which is originated from the interaction between Cp_z and metal d_{z2}, is located above the fermi level (hence below the usual scaling trend line, Figure 5 in main text). On the other hand, the antibonding orbital is partially filled in the case of Cu, making the binding energy of *CHO weaker (2.04 eV) than metals in other groups (0.69, 0.65 and 0.67 eV for Os, Ir and Pt, respectively), hence above the usual trend line. So, the concept used here is qualitatively valid for other SACs as well.

References

1. A. Krasheninnikov, P. Lehtinen, A. S. Foster, P. Pyykkö and R. M. Nieminen, *Phys. Rev. Lett.*, 2009, **102**, 126807.
2. X.-F. ILi, Q.-K. Li, J. Cheng, L. Liu, Q. Yan, Y. Wu, X.-H. Zhang, Z.-Y. Wang, Q. Qiu and Y. Luo, *J. Am. Chem. Soc.*, 2016.
3. B. Hammer and J. Norskov, *Nature*, 1995, **376**, 238-240.
